# First Language Attrition and Dominance: Same Same or Different?

**DOI:** 10.3389/fpsyg.2018.01963

**Published:** 2018-11-06

**Authors:** Barbara Köpke, Dobrinka Genevska-Hanke

**Affiliations:** ^1^Octogone-Lordat (Interdisciplinary Research Unit), University of Toulouse, Toulouse, France; ^2^Department of English, University of Oldenburg, Oldenburg, Germany

**Keywords:** bilingualism, attrition, dominance, reexposure, time scales, stability, context dependence, null subjects

## Abstract

We explore the relationship between first language attrition and language dominance, defined here as the relative availability of each of a bilingual’s languages with respect to language processing. We assume that both processes might represent two stages of one and the same phenomenon (Schmid and Köpke, 2017; Köpke, 2018). While many researchers agree that language dominance changes repeatedly over the lifespan (e.g., Silva-Corvalan and Treffers-Daller, 2015), little is known about the precise time scales involved in dominance shifts and attrition. We investigate these time scales in a longitudinal case study of pronominal subject production by a near-native L2-German (semi-null subject and topic-drop but non-pro-drop) and L1-Bulgarian (pro-drop) bilingual speaker with 17 years of residence in Germany. This speaker’s spontaneous speech showed a significantly higher rate of overt pronominal subjects in her L1 than the controls’ rates when tested in Germany. After 3 weeks of L1-reexposure in Bulgaria, however, attrition effects disappeared and the overt subject rate fell within the monolinguals’ range (Genevska-Hanke, 2017). The findings of this first investigation are now compared to those of a second investigation 5 years later, involving data collection in both countries with the result that after 17 years of immigration, no further attrition was attested and the production of overt subjects remained monolingual-like for the data collections in both language environments. The discussion focuses on the factors that are likely to explain these results. First, these show that attrition and language dominance are highly dependent on immediate language use context and change rapidly when the language environment is modified. Additionally, the data obtained after L1-reexposure illustrate that time scales involved in dominance shift or attrition are much shorter than previously thought. Second, the role of age of acquisition in attrition has repeatedly been acknowledged. The present study demonstrates that attrition of a highly entrenched L1 is a phenomenon affecting language processing only temporarily and that it is likely to regress quickly after reexposure or return to balanced L1-use. The discussion suggests that dominance shift and attrition probably involve similar mechanisms and are influenced by the same external factors, showing that both may be different steps of the same process.

## Introduction

Over the last decades, research on language attrition has progressively become part of the field of bilingual development, together with studies on first language development, second language acquisition and age related changes in language use and/or cognition (see for instance, the chapters in De Bot and Schrauf, 2009). In such a perspective, attrition is often defined as “(…) the loss of language proficiency within an individual over time” (De Bot and Schrauf, 2009, p. 11). In many studies on attrition, researchers seem to take for granted that attrition involves or may even be causally linked to a change in language dominance, which we refer to as *dominance shift* in the following. In the case of attrition of the first language (L1), it is assumed that continuous immersion in a second language (L2) environment will lead to a growing influence of the L2 on the L1, which is then becoming the non-dominant language. In the case of L2 attrition, an individual who was previously immersed in an L2 environment returns to the L1 environment, where the L1 is regaining dominance again (e.g., Hansen, 1999).

The interest in language dominance and in the factors involved in it has considerably increased in recent years (e.g., this issue; Silva-Corvalan and Treffers-Daller, 2015). This is fortunate since previous research often lacked precision with respect to what was meant by language dominance. Furthermore, the link between language dominance and attrition on the one hand, and these two processes and cross-linguistic influence on the other hand, are not understood very clearly. Already in 2004 Köpke and Schmid suggested a relationship between attrition and dominance and hypothesized that “… even if a reversal in language dominance is not necessarily followed by attrition, it is most likely that attrition is preceded by such a reversal …” (2004, p. 12). In the same vein, these authors proposed recently that L1 attrition may “…refer to any of the phenomena that arise in the native language of a sequential bilingual as the consequence of the co-activation of language, cross-linguistic transfer or disuse” (Schmid and Köpke, 2017, p. 637), suggesting a similarity between different processes of interaction between the languages of a bilingual. Such a suggestion is not incompatible with recent conceptions of language dominance. For instance, it has been proposed that language dominance is not a uni-dimensional phenomenon but a complex construct involving a variety of dimensions and remaining relatively independent for different linguistic domains (Birdsong, 2018). This is undoubtedly also the case for attrition. However, we think that it is probably premature at the present stage to conclude that language dominance and attrition refer to one and the same process. In order to examine this question, more data on bilingual development at different points in time during the life of an individual are needed in order to investigate the linguistic changes observable at different time-scales – days, weeks, or years – after modifications in the linguistic environment (including loss of language contact and subsequent reexposure), or other factors (such as attitude changes) that are still poorly understood at the present moment.

With this work, we aim to contribute to a better understanding of the links between dominance shift and L1 attrition. In order to do so, we will first provide a short overview of different possible definitions and operationalizations of the concept of language dominance. We will then focus on the dynamics of changes in language dominance and attrition, through a review of studies focussing on the time scales of these processes in longitudinal studies. Special attention will be paid to the issue of reexposure to a previously attrited (or supposedly attrited) language, a question that has not received much attention until now despite its potential interest for a more comprehensive understanding of the dynamics of dominance shift and attrition as well as the factors that may influence them. We then present the findings from a longitudinal study on subject use in a Bulgarian-German late bilingual tested at four investigation times over a period of 5 years. While data obtained with single case studies are rather limited and generally disallow for generalizations, they represent the type of data crucially needed. Data of this kind lead us to a discussion of the external factors that may explain the effects of reexposure observed and on their relevance for the debate on the links between dominance shift and attrition.

## Language Dominance in Research With Bilingual Speakers

While many studies on bilingualism refer to the concept of language dominance in the description of their participants or discussion of their results, the term itself is, to our knowledge, most of the time not clearly defined. What appears in the use made of the term seems to refer to quite different conceptions of language dominance and further depends on whether the studies focus on bilingual children or adults.

A lot of studies implicitly or explicitly define *dominance* as the relative proficiency in each of the languages of a bilingual (e.g., Silva-Corvalan and Treffers-Daller, 2015). In such a perspective, studies focusing on children most of the time refer to a *strong* and a *weak* language established through production measures such as mean length of utterance (MLU), vocabulary size, or overall number of utterances (De Houwer, 2009). Language dominance in children has furthermore been related to rates of mixing and directionality of cross-linguistic interference (see Unsworth, 2015, for a review). Other studies establish language dominance mainly through the language of the environment. In heritage language research, for instance, many researchers employ the term *dominant language* to refer to the majority language (e.g., Rothman, 2009). Others refer to exposure criteria for each child, as Mayr et al. (2014) who talk about *English-only-homes* vs. *Welsh-only-homes*.

Recently, Unsworth (2015) demonstrated that proficiency and exposure criteria are closely linked in young children in an investigation of 18 Dutch–English bilingual children, aged 2–4 years. Such studies seem to substantiate the claim that exposure is a valid indicator of dominance (in terms of relative proficiency in each language) and has led the author to suggest that exposure can be used as a proxy of language dominance. Others go even further and propose that language dominance is a complex factor, involving proficiency-related components as well as both external (input) and functional (use, context) components (Montrul, 2015; see also de Almeida et al., 2017; Hamann and Abed Ibrahim, 2017). But on the whole, for the developing languages of a bilingual child, relative proficiency in each language seems to be the principal criterion of language dominance adopted in current research.

With respect to adult bilinguals, Wei (2007) referred to a mixture of proficiency and exposure criteria when he proposed that a *dominant language* is the one the bilingual is more proficient in and the one that is used more frequently. However, while for bilingual children the links between frequency of use or input and proficiency are evident in most studies, this is much less straightforward when adult bilinguals are considered. In order to compensate for the absence of such a direct link between use and proficiency in adults, many studies seem to seek to establish linguistic markers of dominance with a large variety of means for establishing relative proficiency (see Flege et al., 2002, for a summary). These include measures of processes involved in utterance planning and lexical access or directionality of code-switching/transfer (Daller, 2011), lexical richness (Treffers-Daller, 2011), discourse patterns (Flecken, 2011), fluency measures, and C-Tests (Daller et al., 2011), among others. The underlying rationale of these studies is similar to what is proposed in studies on children: balanced bilinguals will have similar proficiency measures with respect to various aspects of language use, while speakers who are dominant in one language will achieve higher scores and proficiency measures in that particular language. Thus, in these approaches, *dominance* equals increased proficiency.

However, some authors disagree with the point of view that dominance is mainly an issue of relative proficiency. For instance, Gertken et al. (2014) propose that dominance is independent from proficiency and that it is possible for a speaker to be dominant in a less proficient language. This is in line with a more psycholinguistic definition of dominance, based on the relative availability of each of the languages of a bilingual, as known from studies on lexical retrieval and access.

In a very early study, Lachman and Mistler-Lachman (1976) pursued the question whether an L2 could become dominant over an L1. With reference to models of information processing, they relate language dominance to “the ability to process a language” (p. 282, our translation), the dominant language being the one that is more easily processed. In other terms, in their study focussing on lexical retrieval of single words, they considered the dominant language to be “the language in which the person will retrieve words easier.” They furthermore distinguish the dominant language from what they call the *usual language* which is the language used predominantly. Contrary to most approaches to language dominance, they consider the increased use of the usual language as a necessary but insufficient condition for the establishment of language dominance. Hence, in their study, specific attention was paid to the selection criteria for the participants – they had to have highly predominant use of their L2 (i.e., more than 80% of the language use reported) in order to state that the L2 was the usual language. The authors then investigated whether the usual L2 was also the dominant language of the participants. Six German–English late bilinguals were tested with a timed picture naming task, which was remarkably well-controlled in L1 and L2. Participants were aged between 22 and 48 years and had spoken predominantly English for a period of one to 27 years. The results showed that the participants who had spent less than 7 years in an L2 environment were slower to name pictures in L2 than in L1, while those who had spent 15 or more years^1^ in an L2 environment showed the reverse pattern and were slower to name pictures in L1. While the time scales observed have to be considered with much caution given the limited number of participants, it is worth noting that this study was the very first empirical investigation of L1 attrition reported in the literature (although the term *attrition* was not used) and that the starting point of the authors was to look for a reversal in language dominance patterns.

Similar findings were reported a couple of years later with respect to lexical access in a lexical decision paradigm. Frenck-Mestre (1993) investigated lexical recognition in the L2 of 20 Anglophone undergraduate students attending a French university at the time of testing and showed that skilled bilinguals who had been living in an L2 environment for 3 years or longer, responded faster to L2 words than to L1 words, while beginning bilinguals who had been living in an L2 environment for less than 6 months responded faster in L1. This observation was interpreted as a shift in language dominance illustrating the role of previous experience and actual contact with the language in a word identification experiment.

The findings of these studies suggest an alternative interpretation of the concept of language dominance in terms of *processing facility* or *processing ease* (a label used very recently by Birdsong, 2018, p. 2). In this view, a dominance shift arises as a consequence of increased use of an L2 and leads to a delay in L1 processing. More precisely, the findings of these early studies suggest, in a very preliminary way, that a dominance shift may arise more quickly in perceptual processing, involved in word recognition tasks (3 years in Frenck-Mestre, 1993) than in language production as evidenced by results of the naming task used by Lachman and Mistler-Lachman (1976). Similar observations have been reported by Mägiste (1979) in another early study with 163 bilingual adolescents who showed shorter processing times for L2 after 4–6 years of L2 immersion in comprehension tasks and after 6 years in production tasks (see Köpke and Schmid, 2004, for more details). Moreover, this view assumes that a language (generally the language of the environment) may be more accessible for psycholinguistic processing even though its production does not always equate high-proficiency with respect to phonological, grammatical and even other lexical features (as established with proficiency measures). Such a view is easily implemented in the context of lexical processing and corroborated by recent studies showing that even a few months of immersion in a foreign language, as is typically the case for students in a study abroad program, may lead to increased response times in L1 picture naming (Baus et al., 2013) or to a reduction in lexical retrieval in a verbal fluency task (Linck et al., 2009). Whether processing ease may be at play in a similar way with respect to syntax is less clear yet, but there are studies suggesting that preferences in syntactic online processing may, similarly, be influenced by language context, e.g., with respect to relative clause attachment (Dussias, 2004; Dussias and Sagarra, 2007).

In general, such insights from studies on dominance shift have not sufficiently been taken into account in attrition research – despite the fact that they are perfectly compatible with frequency-based accounts commonly referred to in attrition research, such as the Activation Threshold Hypothesis, ATH (Paradis, 2004; Paradis M., 2007), which predicts that the availability of linguistic material in the bilingual mind will be dependent on frequency and recency of use. However, L1 as well as L2 attrition studies have suggested that attrition cannot be explained by frequency of use alone (see Schmid, 2007 and Mehotcheva and Köpke, 2019, for reviews). Instead, it has been proposed that only a combination of factors may provide the conditions for attrition to arise (see also Schmid and Yilmaz, 2018). Similar perspectives have been taken up with respect to language dominance. It has been proposed that the dominant language is not only the more active language in bilingual processing (and the one related to automaticity), but that it is also influenced by extralinguistic factors such as language attitude for instance (for a review see Gertken et al., 2014). These authors further suggest that dominance may be domain-specific in an individual. This corroborates the idea that bilingual dominance is a complex concept, arising from a combination of criteria (Birdsong, 2014, but also Grosjean, 1998; Flege et al., 2002). Recently, a number of test tools have been proposed that take into account a complexity of this kind. For instance, Dunn and Fox Tree (2009) base their short gradient dominance scale on three main criteria: percentage of use of each language, age of acquisition and age of comfort for both languages. The scale further involves a short question on restructuring of language fluency due to changes in the environment. Gertken et al. (2014) propose a more detailed questionnaire that focuses on language history, use, proficiency and attitudes.

What becomes evident in this review is that dominance shift and attrition are established with similar measures and seem to be influenced by the same factors. Adopting a psycholinguistic approach, it is not unlikely that both processes rely on very similar mechanisms and perhaps represent different stages of a continuum. Following the distinction made by Lachman and Mistler-Lachman (1976), the usual language (as established through frequency of use) will at some point of time become dominant (more readily available for language processing). Whether a dominance change of this kind is equivalent to attrition or whether attrition arises at a later stage of the process, is not clear yet. With respect to L1 attrition in adults, which is the type of attrition the current study focuses on, researchers seem to adopt one or the other standpoint depending heavily on their definition of attrition: if attrition is mainly seen as a phenomenon of on-line processing, dominance and attrition are identical (e.g., Schmid and Köpke, 2017; Schmid and Yilmaz, 2018); if attrition is defined as the restructuring of linguistic representations (e.g., Gürel, 2017; Tsimpli, 2017), then dominance change and attrition are likely to be different and arise at different stages of bilingual development^2^. Köpke (2018) has recently proposed that we may talk about attrition when the processing of the non-dominant language is becoming so cumbersome that disfluencies may be perceived, but there is not much data on perceived attrition at the present moment (be it by the bilingual herself, or by other speakers). So for now, in order to better understand the link between dominance change and attrition, it is probably safer to increase the body of research on the time-scales involved in both processes.

## Time Scales of Dominance Shift and Attrition

In order to investigate the temporariness of dominance shift and attrition processes, we need a clearer picture of the evolution of dominance along the lifespan, reflecting the modifications in exposure and use that arise in a bilingual life. However, to date, the tools that have been developed to establish language dominance do not allow us to capture multiple evolutions across the life-span, despite the efforts that have been made. The short scale proposed by Dunn and Fox Tree (2009) overvalues the acquisition context and attributes a lot of weight to the first acquired language and the question of a possible accent. While the possibility of a loss of fluency is taken up, the single question on this only allows for a binary response. The Bilingual Language Profile by Gertken et al. (2014) is much more detailed with respect to language background and present language use but it doesn’t allow for the consideration of multiple changes in language use either. Thus, none of these tools allows for a satisfactory assessment of multiple dominance shifts and effects of reexposure as they may arise in attrition contexts.

In attrition research, despite consensus about the importance of time, operationalized as Length of Residence (LoR), surprisingly little is known about the time scales involved. Following the rationales of theoretical frameworks as the ATH and memory decay theories, many authors have assumed that L1 attrition in adult speakers is a slow process (Hutz, 2004). In addition, most empirical studies involve participants that have spent at least a decade in their new language environment, as was suggested in the settings of first language attrition studies, namely by Seliger and Vago (1991). However, these studies fail to provide evidence for any direct links between LoR and attrition. In most of them, the observed attrition effects are attributable to a small number of immigrants and are probably due to a complex interaction of multiple factors (e.g., Cherciov, 2013; Opitz, 2013). The number of longitudinal studies providing data on the evolution of attrition over time is still very limited and the conclusions of these studies invite us to revisit the concept of time in relation to attrition. The only group study among them (De Bot and Clyne, 1989, 1994) focused on 40 Dutch immigrants in Australia, who were re-examined 16 years after a first investigation of 200 participants in the early 70s (Clyne, 1981). While the first study suggested that elderly immigrants may suffer from L2 attrition after retirement and reinforce their L1, known as the *language reversion hypothesis*, the second study did not confirm any further changes in these immigrants, neither in L1 nor in L2. This result has been interpreted as evidence for the existence of some kind of a threshold in L2 (and L1?) knowledge, after which the language is no longer sensitive to further changes in use or exposure.

All other longitudinal studies are case studies. Ecke and Hall (2013) report on a study of tip of the tongue (TOT) states in a multilingual subject (five languages) who kept a diary about his TOT states during a period of 10 years. The study focused primarily on the interactions between his most frequently used L3 and L4 (English and Spanish) and his L1 German that was viewed as attriting due to reduced use throughout the study. The results documented the directionality of interference from the more dominant languages to the less dominant L1 and suggest that despite overall high resistance of the L1 to attrition there was a temporary impairment of the L1 in the initial stages of L3 and L4 immersion, where “the overall set of language systems comes out of balance” (p. 1). However, after this period, the L1 gained stability again, suggesting the temporary nature of the phenomenon. Two more studies examined the written production of long-term immigrants (Jaspaert and Kroon, 1992; Hutz, 2004). What all these studies suggest is that the most important changes take place during the first decade of immigration and that longitudinal data, collected after several decades of immersion in a second language environment, do not provide evidence for additional attrition. Given these results, it is surprising that many studies of attrition have continued to focus on immigrants with an LoR of more than 10 or even 15 years.

Some recent studies of L1 processing either in active bilinguals or in second language learners provide more data. For instance, Chang (2012) showed that native speakers of English learning Korean through an intensive language programme provide evidence for temporal changes at segmental, suprasegmental and global levels of pronunciation of their L1 and that this holds even for beginners. In the syntactic domain, Dussias and collaborators showed that previous exposure to specific sentence types may influence relative clause attachment in Spanish-English bilinguals (Dussias et al., 2014; see Schmid and Köpke, 2017, for a more detailed review). These studies suggest that immediate language context may influence language processing and lead to cross-linguistic influence as well as dominance effects on much smaller time scales than previously thought. Again, whether this equals attrition remains an open question. Most importantly, such insights raise the question of the effects of reexposure on the attrited language or a language that has become non-dominant.

However, the question of reexposure is still largely neglected in the field of research on attrition and bilingual development. A small number of studies conducted with adoptees, re-exposed to their native language later in life, mainly focus on the reminiscents of a childhood language and on the benefits of later relearning (e.g., Oh et al., 2019; Pierce et al., 2019). The only longitudinal study in the context of attrition we know of is a study describing dominance shifts in an English-Bulgarian bilingual child (Slavkov, 2015), but given the young age of the child (1;7–2;3) it seems difficult to generalize to what happens in adults.

As far as adult immigrants are concerned, some evidence about reexposure is provided by Stolberg and Münch (2010) who examined a very long term immigrant with no L1 contact at all for 50 years. For the purpose of the study, the participant was interviewed every 2–3 months during a period of 4 years. The data shows a decrease of disfluencies and grammatical errors over time, suggesting that even such a reduced amount of language contact, as in the context of this study, may be sufficient to reactivate a first language. In the phonetic domain, adaptation to VOT standards of one or the other language in bilingual speakers has been shown to be sensitive to immediate linguistic context, a phenomenon called *gestural drift* (Sancier and Fowler, 1997), providing evidence for the immediate effect of reexposure for phonetic aspects.

For the domain of syntax, there are, to our knowledge, only two studies on late bilinguals specifically focusing on reexposure. Chamorro et al. (2016) investigated antecedent preferences for pronominal subjects in Spanish-English bilinguals within the framework of the Interface Hypothesis. They tested two groups of 24 L1 Spanish speakers who had been living in the United Kingdom for a minimum of 5 years. One of these groups had been (re)-exposed to Spanish for at least a week before testing. A control group involved Spanish speakers with very little knowledge of English who had only recently arrived in the United Kingdom (with a mean LoR of 8 weeks). The linguistic material was tested in an offline judgment task and in an online eye-tracking experiment. While there were no differences between the groups in the judgment task, non-exposed attriters showed a lack of online sensitivity for pronoun mismatches in the eye-tracking measures, which distinguished them from both the control group and the recently re-exposed group. Similar results were obtained in a case study on pronominal use in spontaneous speech production by Genevska-Hanke (2017), details follow below. The authors of these studies interpret their results as evidence for the conclusion that attrition affects interface structures without causing permanent changes to knowledge representations (in the sense of language competence) in late bilinguals. The attested changes in attrition are temporary instead and we use the terms *temporary* and *temporariness*, when referring to those in the following. However, as most attrition studies, the two studies are cross-sectional and not longitudinal.

In sum, the picture arising from the literature reviewed here, is that bilingual subjects are sensitive to context of use in a much more immediate fashion than previously thought. However, when and what is influenced by the linguistic context is not yet perfectly clear. All we know is that attrition is most likely to arise “… in those instances where the two languages are sufficiently similar to allow some kind of spillover” (Schmid and Köpke, 2017, p. 653). This is specifically the case for domains where the same linguistic features are present in both languages but are subject to distributional variation of some kind. Since the present investigation was aimed at capturing the evolution of linguistic behavior in L1 at different points of time, we focussed on the alternation of overt vs. null pronominal subjects in speech, a linguistic phenomenon that has previously been shown to be sensitive to variation in different populations (monolinguals, bilinguals, second language users and attriters). Moreover, the languages investigated here, Bulgarian and German, are a promising combination with respect to this phenomenon, as outlined in the next section.

## Linguistic Background

### Previous Research on Overt and Null Pronominal Subjects in L1 Attrition

The alternation of null and overt subjects at the syntax-discourse interface has been investigated for different language combinations in recent research on language attrition. Sorace (2005) tested near-native L2 English speakers with L1 Italian on subject use after prolonged exposure to English. These speakers overproduced overt subjects, performing significantly different from Italian monolinguals in topic continuity contexts (see Tsimpli et al., 2004 for details). The same pattern was also found for L2 speakers of Italian (same language combination) and the attested difficulties have been termed *residual optionality* for L2 speakers and *emerging optionality* for speakers with L1 attrition. This led Sorace to the postulation of the *Interface Hypothesis* as a unified framework of bilingualism, treating L2 acquisition, bilingual L1 acquisition and L1 attrition alike (Sorace and Filiaci, 2006 and related work). According to this hypothesis, phenomena that are purely syntactic (at an internal interface) are impervious to attrition and acquirable in L2, while external interface phenomena might lead to persistent deficits in both groups of speakers. In particular, it is the integration of syntactic and discourse properties at the syntax-discourse interface, which is viewed as problematic. The difficulties of the speakers are attributed to either deficient competence or processing but note that representational accounts do not exclude co-occurring processing deficits. In addition, there is a debate on the role of related cross-linguistic differences. Sorace et al. (2009) suggest that this role is minor, because overproduction of the kind in question has not only been attested for speakers of language combinations of a pro-drop and a non-pro-drop language like Italian-English but also for Spanish-Italian bilinguals, who are speakers of two pro-drop languages. However, there has been recent evidence for differences across languages in relation to the scope of overt pronouns (see Filiaci, 2010 for Spanish vs. Italian and Prentza and Tsimpli (2013) for Spanish vs. Greek). Crucially, the possible impact of cross-linguistic differences on bilinguals’ performance does not exclude but rather enhances co-occurring processing effects.

Looking at more research on pronominal use in L1 attrition, similarly deviant performance has been attested for other language combinations and various interface phenomena (e.g., Tsimpli, 2007; Perpiñán, 2013; Caloi et al., 2018; Di Dimenico and Baroncini, this issue). For instance, Tsimpli (2007) discusses data from two studies on the interpretation and production of postverbal subjects as well as the alternation of null and overt subjects by L1 Greek speakers with a near-native competence of English, Swedish and German^3^. The results revealed that the attrited speakers performed significantly different from the monolingual controls without L1 attrition. Perpiñán (2013) investigated the use of postverbal subjects in *wh*-movement constructions in Spanish, testing the performance of L1 Spanish L2 English bilinguals with postpuberty L1 attrition. No effects were found for postverbal subjects in *wh*-matrix questions (considered purely syntactic) but for the same type of subjects in embedded sentences, in which discourse plays a role (focus interpretation in particular), attrition effects were attested. Caloi et al. (2018) and Di Dimenico and Baroncini (this issue) also investigated the use of postverbal subjects in relation to the realization of new information focus in L1 Italian L2 German speakers, attesting residual optionality in the competence of attrited and heritage speakers of this language combination. Two of the rare attrition studies providing some results on reexposure also focus on the use of null subjects (see Chamorro et al., 2016; Genevska-Hanke, 2017, mentioned above).

### The Two Languages of Investigation – Bulgarian vs. German

Turning our attention to the overt and null subject alternation, in contrast to non-null subject languages like English and semi-null subject languages like German (both non-pro-drop languages), null subject languages like Italian, Greek, Spanish and Bulgarian (all pro-drop or consistent null subject languages) allow for null referential pronominal subjects (labeled *pro*) in addition to overt referential pronominal subjects in finite clauses, giving rise to a pattern of alternation (Bojadziev et al., 1999; Genevska-Hanke, 2019, for Bulgarian; Rizzi, 1986; Jaeggli and Safir, 1989; Roberts and Holmberg, 2010, for the other languages listed above). Note that while the term *pro-drop* refers to a special type of null subjects (*pro*), the term *null subject* is not restricted to a particular type of null subject. Thus, while both German and Bulgarian are null subject languages, only Bulgarian is pro-drop^4^ Examples (1a), (1b), and (1c) illustrate referential subject use in Bulgarian and German. In each case there is reference to one 1PSG subject (as the subject of the main clause) and one 3PSG subject (as the subject of the subordinate clause), both referential and definite. The construction with two overt pronominal subjects given in (1b) represents the only grammatical option in non-pro-drop languages like English. Since spoken German allows for null topic subjects clause-initially, the 1PSG subject is also grammatical, compare (1c), but this is due to null topic licensing by a different grammatical mechanism, termed *topic-drop*. Thus German is also a topic-drop language, but note that topic-drop is restricted to the spoken register (e.g., Hamann, 1996; Haegeman, 2013; Trutkowski, 2016). German null topics are subjects and objects that are only licensed in clause-initial position and further need to be recovered through discourse in the same way Chinese null topics are recovered, see examples (2a)–(2c) from Hamann (1996).


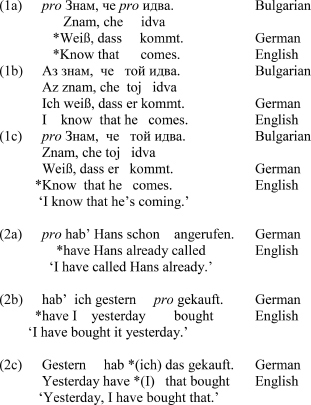


From a cross-linguistic perspective, both languages, Bulgarian and German allow null referential pronominal subjects. However, in Bulgarian these subjects are licensed through pro-drop and are thus unrestricted in their distribution as to clausal position, while in German they are licensed through topic-drop and only occur clause-initially. Furthermore, German null topics are register-dependent and thus a feature of spoken language. Accordingly, overt referential subjects are generally used to a much higher extent in German than they are in Bulgarian so that a possible influence of German would be an increased use of overt subjects^5^. Furthermore and crucially, the overt referential subjects in German overlap with Bulgarian null subjects in contexts of topic continuity – in other words, while German uses overt subjects, Bulgarian uses null subjects in the very same contexts. This further reflects the difference in the scope of overt pronouns between pro-drop and non-pro-drop languages: in the former type of languages overt pronouns carry both *+ topic shift* and *– topic shift* features; in contrast, in the latter type of languages, they only carry a *+ topic shift* feature since null pronouns are associated with the *– topic-shift* feature, giving rise to a one-to-one mapping pattern for overt and null pronouns (Tsimpli et al., 2004). In addition, the less restrictive grammar is taken to affect the more restrictive grammar, so that for speakers of a pro-drop L1 with a dominant non-pro-drop L2, neutralization of native distinctions toward the less restrictive L2 option sets in.

The overt vs. null alternation pattern in pro-drop languages also depends on discourse and is thus not exclusively grammatically-driven. Hence, subject use is generally dependent on conditions of the syntax-discourse interface. While overt referential subjects are predominantly used in focal and topic shift contexts, null referential subjects occur in topic continuity contexts, compare the Italian examples (3) and (4) from Roberts and Holmberg (2010):


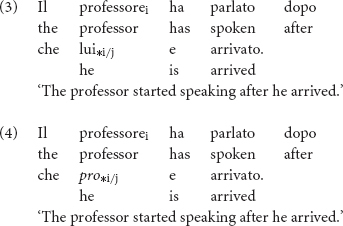


This gives rise to specific patterns that are strongly preferred by native speakers (see Sorace, 2005 for Italian). In other words, these patterns are a matter of preference rather than categorical behavior so that sometimes overt subjects surface in topic continuity or non-focal contexts. This is probably due to the following: on the one side, both types of constructions, one with an overt and one with a null subject are generally possible in pro-drop languages (recall examples 1a and 1b); on the other side, the type of pronouns has to be considered in relation to their scope^6^. As above mentioned, there are cross-linguistic differences as to the scope of overt pronouns in pro-drop languages (see Filiaci, 2010 for Italian vs. Spanish and Prentza and Tsimpli (2013) for Greek vs. Spanish and Di Domenico and Baroncini, this volume for Italian vs. Greek), but despite these it generally holds that non-native speakers with a non-pro-drop L1 use overt subjects to a significantly higher extent than native speakers. This has been also attested for some postpuberty L1 attrition speakers but as recent studies on reexposure suggest, their attrition might be temporary. After all, the difference between overt subject use of native speakers in comparison to that of non-native speakers is one of degree and can be, e.g., directly read off the rates of the overt and null subject alternation for the language under consideration. For Bulgarian, a distribution of 27% of overt and 73% of null pronominal subjects in speech has been attested (Genevska-Hanke, 2017, 2019; see Lorusso et al., 2005, for similar data on Italian and Di Domenico and Baroncini, this issue, for similar data on Italian and Greek)^7^. This information on subject rates is relevant since we use spontaneous speech production in the present study (see also Di Domenico and Baronchini, this issue, for the implementation of similar data).

### Focus of the Present Study

As discussed in detail above, while many researchers agree that language dominance changes repeatedly over the lifespan (e.g., Silva-Corvalan and Treffers-Daller, 2015), studies generally focus on the first shift of language dominance that may arise after emigration and there are hardly any studies that take into account reexposure to a formerly attrited language. Reexposure is possibly neglected, because of the general assumption that L1 attrition in adults is a slow process, arising after decades of non-use, and also because of the difficulty to conduct longitudinal research. But if we want to make the picture of the processes at play in bilingual development more complete, we need more longitudinal data taking into account reexposure to a formerly attrited language.

The aim of the present study is to help modestly fill this gap by means of a detailed examination of the effects of changes in language environment and reexposure. We assume that this will allow us to contribute to a better understanding of the interplay between dominance and attrition since we adopt a psycholinguistic approach considering both dominance shift and attrition as modifications of the availability of linguistic structures for ongoing language processing. The time scales involved in the changes in availability of lexical items have already been documented to a certain extent, while data on similar processes with respect to grammatical structures are crucially needed. We want to know whether sentence processing strategies may show similar sensitivity to language exposure and use, and explore the possible temporariness of these changes.

The present study provides data from a longitudinal study of a late Bulgarian–German bilingual, investigated at four different points of her bilingual development. The focus is on the use of overt and null pronominal subjects that has proved to be sensitive in the context of language contact and bilingual development, recall the attested overproduction. We assume that one should be able to capture even subtle changes in overt vs. null subject alternation patterns after short periods of reexposure. Since the data used here is spontaneous speech production, this will further allow us to add more ecological data to the mostly experimental data obtained in previous studies.

## Materials and Methods

We investigated pronominal use in the spontaneous speech of a bilingual speaker of the language combination L1 Bulgarian L2 German. She is a late bilingual and modifications in her use of pronominal subjects have been attested in a previous study (Genevska-Hanke, 2017). That study focused on the rates of overt and null subjects used by the speaker under consideration of context (topic shift, topic continuity and focal contexts) and aimed at spotting possible overproduction of overt pronominal subjects (see sections Procedure and Review of the Results at Investigation Point 1 for details). Its findings showed that the participant produced significantly more overt pronominal subjects than monolingual Bulgarian speakers in a first investigation, but returned to performance within the monolingual range after 2 weeks of vacation in an L1 environment. In the present study, we aimed at gathering further data on the linguistic trajectory of this bilingual subject and added a second data collection point 5 years later, with another reexposure situation in a follow-up design. The merits of this rather untypical case study are its rare status of being longitudinal, the significant length of L2 exposure in combination with limited L1 contact and the specific language background of a Slavic pro-drop language and a Germanic non-pro-drop but semi-null subject language, additionally allowing for null topics in its spoken register, which has not been studied in the context of L1 attrition so far.

### Participants

Eleven adult Bulgarian native speakers were recorded while conversing, one bilingual speaker and 10 monolingual speakers. The monolingual data is from Genevska-Hanke (2019). We used a questionnaire on language background for all participants, which included questions related to age of initial exposure, language proficiency, duration and extent of language influence, languages of family members and friends as well as to previous and current language use, countries of residence (for the lifespan), schooling and age^8^. In relation to language use, detailed information was gathered as to patterns and extent of use at home, at work, with different conversational partners etc.

The bilingual speaker, who is our test participant, grew up as a monolingual speaker of Bulgarian with the exception of learning English in a school setting from grade 5 to grade 7. Both her L2s, German and English, were acquired after puberty and thus fall into the domain of late second languages. She is a proficient speaker of German as a foreign language (attested by a certificate for the foreign and second language proficiency level C1 of the Common European Framework of Reference) and she majored in sociology in Germany. At investigation point one (IP1), she was 32 years old and has lived in the target language country for 12 years. By the time of this first investigation point, she had extremely limited contact to her native language Bulgarian (short stays in Bulgaria roughly every second year and overall rare contact to the language). According to the analysis of the questionnaire data and according to the criteria for near-nativeness as defined by Tsimpli et al. (2004), her competence in German is considered near-native (see White and Genesee, 1996; Tsimpli et al., 2004 for a definition). In other words, she has reached ultimate attainment of her L2 and her German is hardly distinguishable from that of native speakers without linguistic scrutiny^9^. Following the criteria of the dominance test presented above, including exposure, patterns of use, proficiency and attitudes and on the basis of the answers provided to the questionnaire, German is considered her dominant language. Five years after IP1, there was a second investigation point (IP2) (see Table 1 for details). Three years before this second investigation point, she married a Bulgarian who moved to Germany and started learning German as a second language himself, which strongly affected her daily language use toward a much more balanced pattern of use for the two languages.

**Table 1:** Overview of the recordings of the bilingual.

Investigation point	IP1		IP2	
Recording	1A TC	1B HC	2A TC	2B HC
Country of recording	Germany	Bulgaria	Germany	Bulgaria
Year of recording	2012	2012	2017	2017
Time between recordings	2, 5 weeks		3 weeks	

As for the 10 monolingual speakers of Bulgarian (our control group), all participants are considered predominantly monolingual since they had some limited foreign language instruction at school (several decades before recording), which is typical for people born and raised in Europe in their age (mean age 50, age range 30–67)^10^. All 10 are native speakers of Bulgarian, Bulgarian residents born to Bulgarian monolinguals in Eastern Bulgaria (region of Varna), with no or only vacation stays in foreign countries. All participants had either gained a BA degree or completed professional training after graduating from high school.

In relation to data collection, all subjects gave written informed consent in accordance with the declaration of Helsinki. At the time the research with the monolinguals was planned, the University of Oldenburg did not have a protocol for ethical approval/ethics committee for the humanities. The bilingual speaker gave written consent on anonymity and data handling totally conform to the recommendations of the commission for the evaluation of research consequences and ethics of the Carl-von-Ossietzky University of Oldenburg.

### Procedure

We conducted an exploratory longitudinal study focussing on a single case, compared to a control group. Case studies are particularly indicated in research on the dynamics of developmental processes since these allow the researcher to capture a more fine-grained picture of intra-individual variation over time (e.g., Duff, 2014).

We used spontaneous speech data, which resembles language production in real time. The language of this corpus is informal. Participants were recorded, while conversing with one or more speakers in a naturalistic daily life environment. Each recording lasted 60 min on average. The speakers did not receive any particular instructions prior to the recordings but were informed that the investigation is on the use of Bulgarian in general. In the interviews, they were asked questions thematically linking the conversation to people so that a considerable amount of referential pronouns is used^11^.

The recordings of the monolinguals were produced in 2011 and transcribed, glossed, translated and analyzed thereafter. The ones of the bilingual speaker were produced in 2012 and 2017, see Table 1 for details.

For each speaker of the control group, 200 utterances on average were analyzed with one exception – for one speaker we collected several recordings with a total of 1000 utterances with the aim of increasing the reliability of the data. Four recordings of the test participant with a total of approximately 550 utterances were analyzed, two at investigation point one, after 12 years of residence in Germany and two after 17 years of residence in Germany. At each investigation point, there was one recording in the country of residence (the target language country, TC, Germany) and a second one after a 2 weeks stay in the home country (HC, Bulgaria)^12^. We analyzed 13 recordings of the controls (nine recordings of nine individual speakers with a length of 200 utterances each and four recordings of one speaker with a total of 1000 utterances).

In the analysis, overt and null subjects were calculated per speaker and per clausal type under consideration of subject and context type. All relevant contexts were considered: focal, topic shift, and topic continuity contexts. Imperatives were excluded, while cases of subject doubling entered the count as two overt pronouns, which minimally raises the respective rates accordingly. For overt subjects, we calculated separate rates for all occurrences of overt subject material (including DPs and pronouns) and for overt pronouns only per participant in order to increase comparability across recordings. Note that overproduction of overt subjects would necessarily affect the null subject rates and subject use would then overall fall short of the monolingual standard.

### Predictions

Starting with the results of the two studies involving reexposure data (Chamorro et al., 2016; Genevska-Hanke, 2017), a clear difference between the time before and after reexposure of the non-dominant, attriting language has been reported. Before reexposure, the performance of the attriters was different from that of non-attrited monolinguals (and further comparable to that of second language speakers), as attested in the studies reviewed above (Tsimpli et al., 2004; Sorace, 2005)^13^. After reexposure, the difference between attrited and non-attrited speakers disappeared and the non-dominant language mirrored the so-called “native standard” or “monolingual norm.” Whether this entails another change of dominance remains to be established. In other words, we generally expect attrition effects to be temporary, which entails that the underlying knowledge representations (or language competence in the sense defined above) will not be affected. Thus, on the basis of the results reported in these studies (including these of IP1), we expect attrition effects for the time before reexposure (for the performance of recording 2A TC) for the present study of the second investigation point, IP2. This prediction is also in accordance with assumptions of the Interface Hypothesis on emerging optionality in L1 attrition. However, since this hypothesis does not predict temporariness of the kind reported in Genevska-Hanke (2017) and Chamorro et al. (2016), the nature of the optionality for late L1 attriters possibly needs reconsideration. For the time after reexposure, there are two possibilities – monolingual-like performance due to the increase in accessibility of the language or its dominance as in the case of the second recording of IP1 or performance comparable to that of the first investigation time of IP1, possibly due to the longer period of time of exposure to L2. We prefer the former over the latter possibility, because L1 stability is reached by age 12 (as suggested by Schmid, 2014), because monolingual performance has been also attested for the pronominal alternation at the syntax-discourse interface after reexposure) and since there has been no counter-evidence for attrition effects upon L1 reexposure after comparably long periods of L2 exposure so far (IP2 for our participant lies 17 years after immigration).

## Results

### Review of the Results at Investigation Point 1

We start with a review of the results at IP1 (Genevska-Hanke, 2017). The monolingual group mean for overt pronominal subjects lies by 27% range 16–36%, *SD* = 0.05794, the group is normally distributed, according to the statistical analysis carried out.

As for the bilingual participant, at the time of the first recording 1A TC, we found overproduction of overt pronominal subjects in the language data of the test participant^14^. Examples (5) and (6) illustrate her use of overt pronominal subjects in topic continuity contexts. Note that the 3PSG subjects in (5) and the 1PSG subjects in (6) all refer to a continuous topic each, established in previous discourse.


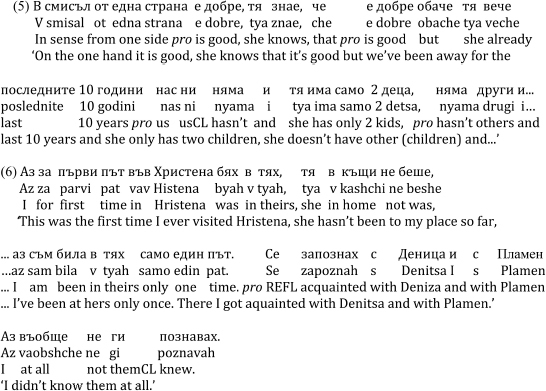


The overall rate of overt pronominal subjects of the bilingual reached 41%, exceeding the upper limit of the non-attrited monolinguals’ rate range. This rate is significantly different from the rates of the controls, two-tailed probability *p* = 0.043, estimated percentage of normal population falling below individual’s score = 99.57% (single case statistics significance test on difference between individual’s score and control, Crawford and Garthwaite, 2002).

However, the statistical analysis of the two separate rates, the 1A TC and the 1B HC rate of the attrited speaker, revealed that these two rates are significantly different. The 1A TC recording rate indicates that overt pronominal subjects are used in up to 47% of all cases and it is significantly higher than the controls’ mean rates, two-tailed probability *p* = 0.009, estimated percentage of normal population falling below individual’s score = 97.85% (same statistical procedure as above, Crawford and Garthwaite, 2002). For the recording 1B HC, there was no overproduction of overt pronominal subjects. The overt pronominal subjects rate was 34%, which lies within the range of the controls, and thus shows comparable performance, two-tailed probability *p* = 0.260, estimated percentage of normal population falling below individual’s score = 86.99% (same type of significance test as above, Crawford and Garthwaite, 2002).

**FIGURE 1:**
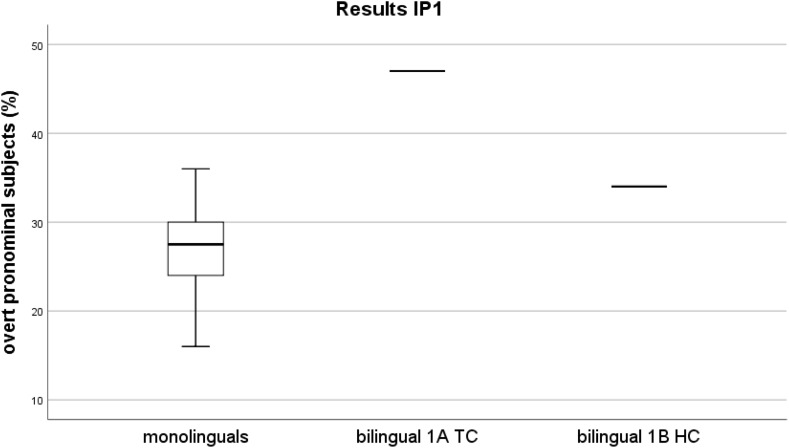
IP1 – Distribution of overt pronominal subjects in percentages. Mean rates over the sum of subjects per recording (*y*-axis). Participants (*x*-axis) – box on the left represents the rates of the monolinguals, horizontal lines to the right indicate the rates of the bilingual for IP1 (line in the middle corresponds to the rate of the 1A TC recording, line on the right corresponds to the one of the 1B HC recording).

Figure 1 illustrates the distribution of rates for the L1 group of controls and the mean rates of both recordings of the speaker with L1 attrition at IP1, 1A TC and 1B HC.

### Results at Investigation Point 2

For investigation point 2, we are using the results of the control group that were already presented in the previous section. The totals of the control group and the 4 rates of the bilingual for both investigation points are displayed in Table 2.

**Table 2:** Distribution of overt and null (pronominal) subjects for IP1 vs. IP2.

	Monolinguals (totals)	Bilingual IP1	Bilingual 1A TC	Bilingual 1B HC	Bilingual IP2	Bilingual 2A TC	Bilingual 2B HC
Number of utterances	2909	249	138	111	229	119	110
Number of subjects	3439	291	163	128	266	132	134
Overt subjects	39%	46%	51%	39%	35%	36%	34%
Null subjects	61%	54%	49%	61%	65%	64%	66%
Overt pronominal subjects	27%	41%*	47%*	34%	27%	29%	24%
Null pronominal subjects	73%	59%	53%	66%	73%	71%	76%

*Significant difference to monolingual group.

*Left most column displays the split to number of utterances, subjects, overt and null (pronominal) subjects (all overt vs. overt pronominal only and their null counterparts). Top line indicates the participants’ mean rates per recording.*

At IP2, no overproduction of overt pronominal subjects was attested, neither in the 2A TC recording, nor in the 2B HC recording. Hence the overall rate of both recordings also falls within the monolinguals’ range (two-tailed probability for 2A TC *p* = 0.710, estimated percentage of normal population falling below individual’s score = 64.29%; for 2B HC, *p* = 0.670, estimated percentage of normal population falling below individual’s score = 33.39%).

The major difference to the performance at IP1 is the fact that this time both rates of overt pronominal subjects, the 1A TC rate of 29% and the 1B HC rate of 24% fall into the non-attrited monolinguals’ rate range. The 1A TC rate is higher than the 1B HC rate, so that this can be interpreted as a similar tendency of a rate drop after reexposure, comparable to that of investigation point one. However, both rates neither differ significantly from one another, nor from those of the monolingual control group (same statistical analyses as those at IP1).

**FIGURE 2:**
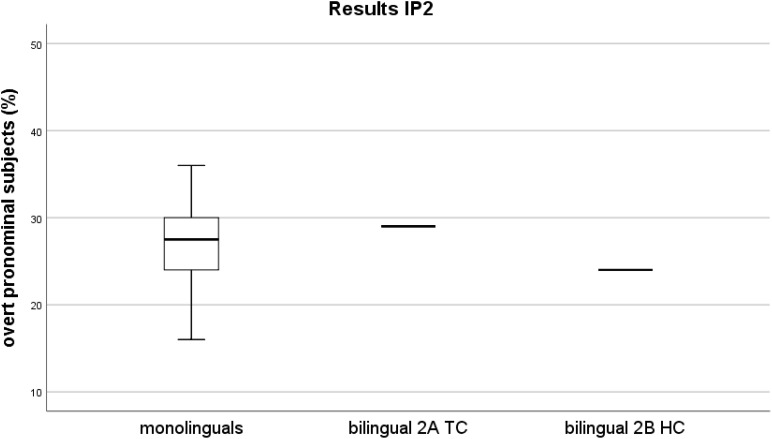
IP2 – Distribution of overt pronominal subjects in percentages. Mean rates over the sum of subjects per recording (*y*-axis). Participants (*x*-axis) - box on the left represents the rates of the monolinguals, horizontal lines to the right indicate the rates of the bilingual for IP2 (line in the middle corresponds to the rate of the 2A TC recording, line on the right corresponds to the one of the 2B HC recording).

**FIGURE 3:**
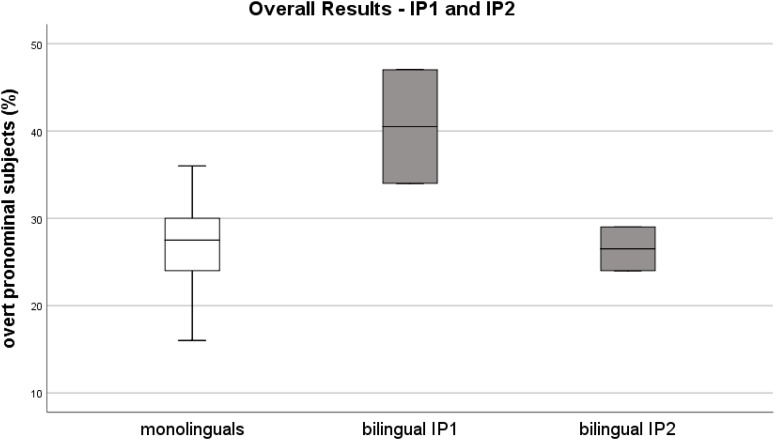
IP1 vs. IP2 – Distribution of overt pronominal subjects in percentages. Mean rates over the sum of subjects per recording (*y*-axis). Participants (*y*-axis) - box on the left represents the monolinguals’ rates, box in the middle those of the bilingual at IP1, box on the right those of the bilingual at IP2.

Figure 2 illustrates the distribution of rates for the L1 group of controls and the mean rates of both recordings of the bilingual speaker at IP2, 2A TC and 2B HC. Figure 3 illustrates the comparison of the monolingual group and the bilingual speaker at both investigation points.

## Discussion

The overall mean rate for overt pronominal subjects of the L1 Bulgarian L2 German bilingual speaker at the first investigation point (IP1) revealed significant overproduction of these subjects. This is similar to what has been observed and interpreted as attrition in other studies investigating pronominal use in bilinguals, recall the details given in section Previous Research on Overt and Null Pronominal Subjects in L1 attrition. However, the corresponding overall mean rate 5 years later, at IP2, indicates that the production of overt pronominal subjects differed no longer significantly from the control group data after 17 years of immigration. Additionally, while the overt subject rate of the first recording in the target country (1A TC) was significantly different from the rates of the monolingual controls (which yielded a further difference for the overall rate of IP1), the overt subject rate of the second recording in the target country (2A TC) was similar to the monolinguals’ rates. Thus, contrary to our expectations, no significant differences were measured and accordingly no optionality was attested at this second investigation point after 17 years of immigration, be it in the target or in the home country.

These findings, first of all, point toward the temporariness of attrition phenomena – very similar to what may be observed with respect to dominance shift. The home country recording of each investigation point was made only after few weeks of reexposure to the native language in the home country and the rate of overt pronominal subjects was lower than that of the target country recording each time (albeit non-significantly for the second investigation point), suggesting that a limited amount of extensive exposure to the L1 is sufficient to return to performance within the monolingual speakers’ range. While the present data have been obtained with only one bilingual subject, they are perfectly in line with the findings of the cross-sectional study by Chamorro et al. (2016) showing that a group of Spanish immigrants in Great Britain who were immersed during 1 week in an L1 environment performed conform to the native standard, whereas a similar group of immigrants in an L2 environment did not. This means that temporariness in attrition phenomena has now been demonstrated in both longitudinal and cross-sectional data. However, and as above discussed, temporariness in attrition is not predicted by the Interface Hypothesis, which suggests that it still has to be accommodated.

Taken together with evidence from the literature reviewed above, this suggests that peculiarities of performance observed in L1 attrition are probably depending much more on language mode and activation states than on restructuring of linguistic representations (see also Schmid and Köpke, 2017). A processing account for modifications in pronoun use has already been proposed by Gürel (2004). In a study on interpretation of null and overt subject pronouns in embedded clauses in Turkish by Turkish-English bilingual immigrants in North America, she showed that cross-linguistic influence was observed only in those cases, in which Turkish and English allow for similar options in the interpretation of pronouns. Gürel explained this finding with reference to the ATH (Paradis, 1993) predicting that the more frequently used option will be activated more easily when two structures (or lexical items) are in competition, but not when there is no competition because the attriting language has no corresponding structure (as for example in the case of a language with grammatical gender in competition with English, e.g., Bergmann et al., 2015). If the phenomena most commonly observed in attrition studies are due to competition of linguistic options that continue to co-exist in the grammar of the speaker, this clearly means that no structural or representational changes are involved and that processing restrictions may be a promising explanation for the obtained results.

Two factors, however, seem to play a major role in the observed temporariness of preferential processing strategies: immediate language background or language context and age of acquisition. We will discuss these two factors in what follows.

The influence of language context has been demonstrated in the data at two levels. First of all, three of the four recordings show an effect of the country where the data collection took place. While the first recording in Germany showed significant deviance from the native norms in Bulgarian, data recorded in Bulgaria were within the native range at both investigation points. Such an influence of the immediate language context on performance in a language has been demonstrated repeatedly in recent studies discussed above (e.g., Chang, 2012; Baus et al., 2013; Dussias et al., 2014). It has even been shown that the manipulation of immediate language context has an influence on nonverbal cognitive skills such as control of cognitive interference (Wu and Thierry, 2013). However, the present study also shows that the immediate language environment involves more than just the country of recording: at IP2, performance of the participant in Bulgarian was within the native range also for the recording done in Germany. This can be explained by a change of language use at home. Recall that the participant married a native speaker of Bulgarian 3 years before this investigation point, which made her shift from a quasi exclusive use of German (IP1) to a much more balanced use of both languages (IP2) in her daily life in Germany. The fact that the use of overt and null pronouns in L1 is once again within the native range at this point, after 17 years of immigration, but along with a balanced use of both languages, emphasizes the temporariness of L1 attrition phenomena for late bilinguals. Taken together, the present study contributes to a more general picture suggesting that the language environment must be considered at macro- as well as micro-levels, including, among others, the country of investigation and the specific personal environment at time of investigation at the macro-level (e.g., did the participant receive visits from L1-speakers in the weeks preceding the investigation?) and the languages of the experimenter and the linguistic setting of the task at the micro-level (Wu and Thierry, 2010; Dussias et al., 2014).

However, the language environment is probably not the only factor of influence. What our findings also suggest is that the attrited language of postpuberty L2 speakers may be reactivated relatively fast; within few weeks only. As previously shown, age of acquisition of the L2 is a major factor in determining qualitative and quantitative aspects of attrition (Schmid, 2014). However, for the moment we can only speculate on the role played by age of acquisition with respect to the effects of reexposure. Crucially, studies on reexposure to an attrited language in early bilinguals that could shed further light on the possible temporariness of attrition in younger bilinguals, are not yet available, except for some studies on language relearning in international adoptees (see Oh et al., 2019, for a summary). Nevertheless, there is sufficient evidence for both the observation that dominance changes are frequent and fast in young children (e.g., Slavkov, 2015), and that they are much slower in adults as suggested by the early studies on dominance shift discussed above, reporting periods of L2 immersion of 3–7 years, depending on the tasks used, for adults (Lachman and Mistler-Lachman, 1976; Frenck-Mestre, 1993) and even adolescents (Mägiste, 1979). However, we have to consider two more dimensions. The type of linguistic knowledge involved matters: these studies concern mainly lexical identification and retrieval processes and it is likely that the time scales involved in dominance change will vary for different types of linguistic knowledge, similar to what has been proposed recently with respect to “critical periods” or other age effects (Birdsong, 2018). But if we assume – as do most studies on language attrition – that the lexicon is most vulnerable to attrition, attaining dominance in L2 for grammatical processing should take even longer than the 3–7 years period observed for lexical processing. Moreover, we have to take into account that these data concern a shift from L1 to L2 dominance, and show that gaining dominance in an L2 over a firmly entrenched L1 takes several years. The present study involves reexposure to the L1 in a late bilingual, and shows that few weeks of immersion are sufficient – if not to reverse dominance – then to at least establish balanced bilingual performance with respect to the grammatical feature investigated here. Hence, for late bilinguals, language status (L1 or L2) is most likely to be a major factor in determining processing ease and permeability to cross-linguistic influence: what seems to remain problematic in L2 acquisition for years (overproduction of overt subjects has been repeatedly attested for L2, as discussed above), may be reestablished within very short time scales after reexposure to a strongly entrenched L1. This points to the fact that different developmental processes as L2 acquisition and L1 attrition need to be considered as distinct, contrary of what is predicted, for instance, by the Interface Hypothesis.

Now, what about our initial question concerning the relationship between dominance shift and attrition? Even though the present study did not focus on dominance as such (only the L1 was investigated), the findings presented here stress the temporary nature of cross-linguistic influence as observed in attrition, affecting language processing and depending on a complex interaction of language exposure and use on the one side and language status as determined by age and order of acquisition on the other side. Furthermore, there is ample evidence that L1 attrition in late bilinguals is generally a processing issue (e.g., discussions in Köpke and Schmid, 2011; Schmid and Köpke, 2017). This provides empirical underpinnings to the idea that attrition and dominance shift are very similar, if not identical processes, involving quantitative but not qualitative differences, which strengthens the idea that we may talk about attrition when the availability of the non-dominant language decreases so much that fluent language processing is becoming more and more difficult (Köpke, 2018). This is obviously not necessarily the case in a bilingual who shows increased use of overt pronominal subjects, as in the present study, unless the person additionally experiences disfluencies in language processing or feels insecure about her language use. The reliance on a processing strategy frequently used in the more dominant language may even be a way to avoid disfluencies in the non-dominant language, a strategy also used by L2 learners (recall that overt pronouns in non-pro-drop languages correspond to both overt and null subjects in pro-drop languages in terms of formal overlap). Further empirical studies of dominance shift at different time scales and in different types of bilinguals are called for in order to challenge these hypotheses, and specifically to investigate whether dominance shift and attrition may be considered as different steps of the same process. An ultimate answer to such a complex question will of course need to be discussed very largely within the field.

## Conclusion

The findings of the present study demonstrate the temporariness of attrition phenomena in the domain of pronominal subject use at the syntax-discourse interface. This can be interpreted as evidence for the overall stability of a fully-developed L1 in a late bilingual, as previously proposed for L1 attrition (e.g., Schmid, 2014). Furthermore, L1 attrition in late bilinguals is most likely to arise due to competition of related processing strategies, similar to what definitions of language dominance that go beyond the relative proficiency in each language suggest (Gertken et al., 2014). Viewed in this way, attrition effects appear to be very sensitive to immediate language context at both the macro- and the micro-level. The time scales involved are further dependent on the degree of entrenchment of the language, influenced by age of acquisition of the L2 and the status of the language under investigation (first or second language) to a notable extent.

Thirty years of attrition research have demonstrated very clearly that language systems are dynamic and sensitive to language context. While cross-linguistic influence remains restricted to specific linguistic domains and never entails high error rates, it seems to arise very early in the language contact process, at least as far as specific linguistic structures are concerned. This needs to be investigated and documented carefully in future research employing processing measures under consideration of different types of time scales as well as various settings of immersion and reexposure. Research on language dominance seems a promising way to do this.

## Author Contributions

BK developed the main ideas of the manuscript and wrote the sections Introduction, Language Dominance in Research With Bilingual Speakers, Time Scales of Dominance Shift and Attrition, Discussion, and Conclusion. DG-H designed and carried out the study, analyzed its results and wrote the sections Linguistic Background, Materials and Methods, and Results with the exception of the section Focus of the Present Study and parts of the section Procedure. Both authors wrote the abstract, contributed in data interpretation and critically read the manuscript.

## Conflict of Interest Statement

The authors declare that the research was conducted in the absence of any commercial or financial relationships that could be construed as a potential conflict of interest.

## Footnotes

1 But note that there were no participants in between these two values.

2 Please note that this is very close to the continuum between performance and competence in attrition, proposed by Sharwood Smith (1983, p. 51) in the very beginnings of attrition research.

3 See Tsimpli et al. (2004) for details.

4 German is classified as a semi-null subject language, since it requires obligatory null non-argumental expletive subjects in all non-clause-initial positions, independent of register (Biberauer, 2010; Roberts and Holmberg, 2010).

5 Note that we do not completely exclude the possibility that the presence of null topics in German influences bilinguals’ performance so that there might be less overproduction of overt subjects in comparison to the case of a non-null subject language like English. Since null topics overlap with null subjects in pro-drop languages in clause-initial contexts, a “non”-difference in subject use between clause-initial and non-clause-initial contexts would be revealing – in pro-drop languages, null subject rates are higher for subordinate clauses, which in combination with clause-initial topic-drop would give rise to a pattern of a more balanced use of null subjects in clause-initial and non-clause-initial contexts. However, this was not attested in our data. This might be further influenced by the fact that topic-drop is a phenomenon of spoken German. Furthermore, a study on near-native L1 Bulgarian L2 German speakers revealed that topic-drop was not acquired so that these speakers transferred the null subjects of their pro-drop L1 to their L2 (Genevska-Hanke, 2019).

6 Cardinaletti (2004) discusses an increase of overt subject use in Italian (see also Frascarelli, 2007 for related evidence from corpora on spoken Italian), suggesting that the paradigm and the status of Italian personal pronouns are undergoing a change from “strong” to possibly “weak” in the sense of Cardinaletti and Starke (1994). For instance, the 3rd person *lui* and *lei* are already considered weak and are slowly taking up the slots of the archaic *egli* and *ella*. Since the null pronoun *pro* is also weak, the overt weak form can freely alternate with *pro*, without violating grammar.

7 The rates are from Genevska-Hanke (2019), details follow below.

8 The questionnaire was developed on the basis of the NOWETAS adaptation of the Beirut-Tours Questionnaire on child bilingualism (Paradis J., 2007). It is available in Bulgarian and German, but see the Supplementary Material for a list of selected questions in English. For the bilingual, the questionnaire was applied at each investigation point, prior to and after reexposure.

9 With the exception of foreign accent, to which native speakers are very sensitive, see e.g., Tsimpli et al. (2004) and White and Genesee (1996).

10 Instruction was in Russian, except for one speaker who had English instead due to a change in the schooling system.

11 Possible questions were: “Tell me about your brother…” and “Did you visit anybody lately?”

12 The results of IP1 have already appeared in Genevska-Hanke (2017).

13 There is also a study on pronominal use in L1 Bulgarian L2 German speakers, which provides evidence that near-native speakers’ competence falls short of the native standard (Genevska-Hanke, 2019). This study was not reviewed above since it is an L2 and not an L1 attrition study.

14 Note that the rates of overt subjects in topic shift contexts of the bilingual were comparable across recordings so that the difference between overt subject rates originates solely due to an increased use of overt subjects in topic continuity contexts. This is in line with previous studies, reporting overproduction of overt subjects in precisely these contexts (see section Previous Research on Overt and Null Pronominal Subjects in L1 Attrition for related information and references).

## References

[B1] Baus C. Costa A. Carreiras M. (2013). On the effects of second language immersion on first language production. *Acta Psychol.* 142 402–409. 10.1016/j.actpsy.2013.01.010 23435116

[B2] Bergmann C. Meulman N. Stowe L. A. Sprenger S. A. Schmid M. S. (2015). Prolonged L2 immersion engenders little change in morphosyntactic processing of bilingual natives. *Neuroreport* 26 1065–1070. 10.1097/WNR.0000000000000469 26509547

[B3] Biberauer T. (2010). “Semi null-subject languages, expletives and expletive pro reconsidered,” in *Parametric Variation: Null Subjects in Minimalist Theory*, eds Biberauer T. Holmberg A., I. Roberts, and M. Sheehan (Cambridge: CUP), 153–199.

[B4] Birdsong D. (2014). Dominance and age in bilingualism. *Appl. Linguist.* 35 374–392. 10.1093/applin/amu031

[B5] Birdsong D. (2018). Plasticity, variability and age in second language acquisition and bilingualism. *Front. Psychol.* 9:81. 10.3389/fpsyg.2018.00081 29593590 PMC5857581

[B6] BojadzievT.KuzarovI.PenchevJ. (1999). * 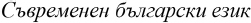 . Modern Bulgarian Language.* Sofia: Petar Beron.

[B7] Caloi I. Belletti A. Poletto C. (2018). Multilingual competence influences answering strategies in Italian-German bilinguals. *Front. Psychol.* 9:1971 10.3389/fpsyg.2018.01971 PMC6220047 30429806

[B8] Cardinaletti A. (2004). “Towards a carthography of subject positions,” in *The Structure of CP and IP*, ed. Rizzi L. (New York, NY: OUP), 115–165.

[B9] Cardinaletti A. Starke M. (1994). The typology of structural deficiency: on the three grammatical classes. University of Venice. *Work. Pap. Linguist.* 4 41–109.

[B10] Chamorro G. Sorace A. Sturt P. (2016). What is the source of L1 attrition? The effect of recent L1 re-exposure on Spanish speakers under L1 attrition. *Bilingualism* 19 520–532. 10.1017/S1366728915000152

[B11] Chang C. B. (2012). Rapid and multifaceted effects of second-language learning on first-language speech production. *J. Phon.* 40 249–268. 10.1016/j.wocn.2011.10.007

[B12] Cherciov M. (2013). Investigating the impact of attitude on first language attrition and second language acquisition from a dynamic systems theory perspective. *Int. J. Biling.* 17 716-733.

[B13] Clyne M. (1981). Second generation foreigner talk in Australia. *Int. J. Soc. Lang.* 28 69–80. 10.1515/ijsl.1981.28.69

[B14] Crawford J. R. Garthwaite P. H. (2002). Investigation of the single case in neuropsychology: confidence limits on the abnormality of test scores and test score differences. *Neuropsychologia* 40 1196–1208. 10.1016/S0028-3932(01)00224-X 11931923

[B15] Daller H. (2011). The measurement of bilingual proficiency: introduction. *Int. J. Biling.* 15 123–127. 10.1177/1367006910380036 26304219

[B16] Daller M. H. Yildiz C. de Jong N. H. Kan S. Basbagi R. (2011). Language dominance in Turkish-German bilinguals: methodological aspects of measurements in structurally different languages. *Int. J. Biling.* 15 215–236. 10.1177/1367006910381197

[B17] de Almeida L. Ferré S. Morin E. Prévost P. dos Santos C. Tuller L. (2017). Identifcation of bilingual children with specific language impairment in France. *Linguist. Approaches Biling.* 7 331–358. 10.1075/lab.15019.alm 24508158

[B18] De Bot K. Clyne M. (1989). Language reversion revisited. *Stud. Second Lang. Acquis.* 11 167–177. 10.1017/S0272263100000590

[B19] De Bot K. Clyne M. (1994). A 16-year longitudinal study of language attrition in dutch immigrants in Australia. *J. Multiling. Multicult. Dev.* 15 17–28. 10.1080/01434632.1994.9994554

[B20] De Bot K. Schrauf R. (2009). *Language Development over the Lifespan.* New York, NY: Routledge.

[B21] De Houwer A. (2009). *Bilingual First Language Acquisition.* Clevedon: Multilingual Matters.

[B22] Duff P. (2014). Case study research on language learning and use. *Annu. Rev. Appl. Linguist.* 34 233–255. 10.1017/S0267190514000051

[B23] Dunn A. Fox Tree J. (2009). A quick, gradient bilingual dominance scale. *Bilingualism* 12 273–289. 10.1017/S1366728909990113

[B24] Dussias P. E. (2004). Parsing a first language like a second: The erosion of L1 parsing strategies in Spanish-English Bilinguals. *Int. J. Biling.* 3 355–371. 10.1177/13670069040080031001

[B25] Dussias P. E. Perrotti I. Brown M. Morales L. (2014). “Re-learning to parse a first language: the role of experience in sentence comprehension,” in *Proceedings of the 27th CUNY Conference on Human Sentence Processing*, Columbus, OH).

[B26] Dussias P. E. Sagarra N. (2007). The effect of exposure on syntactic parsing in Spanish-English bilinguals. *Bilingualism* 10 101–116. 10.1017/S1366728906002847

[B27] Ecke P. Hall C. J. (2013). Tracking tip-of-the-tongue states in a multilingual speaker: Evidence of attrition or instability in lexical systems? *Int. J. Biling.* 17 734–751. 10.1177/1367006912454623

[B28] Filiaci F. (2010). “Null and overt subject biases in Spanish and Italian: a cross-linguistic comparison,” in *Proceedings of the 12th Hispanic Linguistics Symposium*, eds Borgonovo C. Espa M.ñol-Echevarría, and P. Prévost (Somerville, MA: Cascadilla), 171–182.

[B29] Flecken M. (2011). Assessing bilingual attainment: macrostructural planning in narratives. *Int. J. Biling.* 15 164–186. 10.1177/1367006910381187

[B30] Flege J. E. Mackay I. R. A. Piske T. (2002). Assessing bilingual dominance. *Appl. Psycholinguist.* 23 567–598. 10.1017/S0142716402004046

[B31] Frascarelli M. (2007). Subjects, topics and the interpretation of referential pro. An interface approach to the linking of (null) pronouns. *Nat. Lang. Linguist. Theory* 25 691–734. 10.1007/s11049-007-9025-x

[B32] Frenck-Mestre C. (1993). Use of orthographic redundancies and word identification speed in bilinguals. *J. Psycholinguist. Res.* 22 397–410. 10.1007/BF01074343

[B33] Genevska-Hanke D. (2019). Subject Realization in Bulgarian: Overt and Null Subjects in Bulgarian-German Interlanguage. Doctoral thesis, University of Oldenburg, Oldenburg.

[B34] Genevska-Hanke D. (2017). “Intrapersonal variation in late L1 attrition and its implications for the competence/performance debate,” in *Linguistik im Nordwesten: Beiträge zum 8. Nordwestdeutschen Linguistischen Kolloquium*, eds Levkovych N. Urdze A. (Bochum: Brockmeyer).

[B35] Gertken L. M. Amengual M. Birdsong D. (2014). “Assessing language dominance with the bilingual language profile,” in *Measuring L2 Proficiency: Perspectives from SLA*, eds Leclercq P. Edmonds A. Hilton H. (Bristol: Multilingual Matters), 208–225.

[B36] Grosjean F. (1998). Transfer and language mode. *Bilingualism* 1 175–176. 10.1017/S1366728998000285

[B37] Gürel A. (2004). Selectivity in L2-induced L1 attrition: a psycholinguistic account. *J. Neurolinguist.* 17 53–78. 10.1016/S0911-6044(03)00054-X

[B38] Gürel A. (2017). Is every bilingual an attriter?: The unbearable complexity of defining L1 attrition. *Linguist. Approaches Biling.* 7 696–699. 10.1075/lab.00007.gur

[B39] Haegeman L. (2013). The syntax of registers: diary subject omission and the privilege of the root. *Lingua* 130 88–110. 10.1016/j.lingua.2013.01.005

[B40] Hamann C. (1996). Null arguments in German child language. *Lang. Acquis.* 5 155–208. 10.1207/s15327817la0503_1

[B41] Hamann C. Abed Ibrahim L. (2017). Methods for identifying specific language impairment in bilingual populations in Germany. *Front. Commun.* 2:16. 10.3389/fcomm.2017.00016 29790243

[B42] Hansen L. (1999). *Second Language Attrition in Japanese Contexts.* Oxford: Oxford University Press.

[B43] Hutz M. (2004). “Is there a natural process of decay? A longitudinal study of language attrition,” in *First Language Attrition: Interdisciplinary Perspectives On Methodological Issues*, eds Schmid M. S. Köpke B. Keijzer M. Weilemar L. (Amsterdam: John Benjamins), 189-206.

[B44] Jaeggli O. Safir K. (1989). *The Null Subject Parameter.* Dordrecht: Foris 10.1007/978-94-009-2540-3

[B45] Jaspaert K. Kroon S. (1992). “From the typewriter of A.L.: a case study in language loss,” in *Maintenance and Loss of Minority Languages*, eds Fase W. Jaspaert K. Kroon S. (Amsterdam: John Benjamins), 137–147. 10.1075/sibil.1.11jas

[B46] Köpke B. (2018). “First language attrition : from bilingual to monolingual proficiency?,” in *The Cambridge Handbook of Bilingualism*, eds Houwer A. De Ortega L. (Cambridge: Cambridge University Press).

[B47] Köpke B. Schmid M. S. (2004). “First language attrition: The next phase,” in *First Language Attrition: Interdisciplinary Perspectives on Methodological Issues*, eds Schmid M. S. Köpke B. Keijzer M. Weilemar L. (Amsterdam: John Benjamins), 1–43.

[B48] Köpke B. Schmid M. S. (2011). L’attrition de la première langue en tant que phénomène psycholinguistique. *Lang. Interact. Acquis.* 2 197–220. 10.1075/lia.2.2.02kop

[B49] Lachman R. Mistler-Lachman J. (1976). Dominance lexicale chez les bilingues. *Bull. Psychol.* 15 281–288.

[B50] Linck J. A. Kroll J. F. Sunderman G. (2009). Losing access to the native language while immersed in a second language. *Psychol. Sci.* 20 1507–1515. 10.1111/j.1467-9280.2009.02480.x 19906121 PMC2858781

[B51] Lorusso P. Caprin C. Guasti M. (2005). “Overt subject distribution in early Italian children,” in *A Supplement to the Proceedings of BUCLD*, eds Brugos A. Clark M.-Cotton, and S. Ha (Somerville MA: Cascadilla Press).

[B52] Mägiste E. (1979). The competing language systems of the multilingual: a developmental study of decoding and encoding processes. *J. Verbal Learn. Verbal Behav.* 18 79–89. 10.1016/S0022-5371(79)90584-X

[B53] Mayr R. Jones D. Mennen I. (2014). “Speech Learning in bilinguals: consonant cluster acquisition,” in *Advances in the Study of Bilingualism*, eds Thomas E. M. Mennen I. (Bristol: Multilingual Matters), 3–24.

[B54] Mehotcheva T. Köpke B. (2019). “L2 attrition,” in *The OUP Handbook of Language Attrition*, eds Schmid M. S. K B.öpke (Oxford: OUP).

[B55] Montrul S. (2015). “Dominance and proficiency in early and late bilingualism,” in *Language Dominance in Bilinguals*, eds Silva-Corvalan C. Treffers-Daller J. (Cambridge: CUP), 15–35.

[B56] Oh J. Au T.-K. Jun S.-A. Lee R. (2019). “Childhood language memory in adult heritage language (re)learners,” in *The Oxford Handbook on Language Attrition*, eds Schmid M. S. K B.öpke (Oxford: Oxford University Press).

[B57] Opitz C. (2013). A dynamic perspective on late bilinguals’ linguistic development in an L2 environment. *Int. J. Biling.* 17 701–715. 10.1177/1367006912454621

[B58] Paradis M. (1993). Linguistic, psycholinguistic, and neurolinguistic aspects of “interference” in bilingual speakers: the activation threshold hypothesis. *Int. J. Psycholinguist.* 9 133–145.

[B59] Paradis J. (2007). *The Alberta Language Environment Questionnaire (ALEQ).* Available at: https://www.ualberta.ca/linguistics/cheslcentre/questionnaires

[B60] Paradis M. (2004). *A Neurolinguistic Theory of Bilingualism.* ]Amsterdam: John Benjamins 10.1075/sibil.18

[B61] Paradis M. (2007). “L1 attrition features predicted by a neurolinguistic theory of bilingualism,” in *Language Attrition. Theoretical Perspectives*, eds Köpke B. Schmid M. S. Keijzer M. Dostert S. (Amsterdam: John Benjamins), 121–134.

[B62] Perpiñán S. (2013). “Optionality in bilingual native grammars,” in *First Language Attrition*, eds Schmid M. S. K B.öpke (Amsterdam: John Benjamins), 127–156.

[B63] Pierce L. Genesee F. Klein D. (2019). “Langauge loss and language learning in internationally adopted children: evidence from behaviour and the brain,” in *The Oxford Handbook on Language Attrition*, eds Schmid M. S. K B.öpke (Oxford: Oxford University Press).

[B64] Prentza A. Tsimpli I. M. (2013). On the optionality in L2 pronominal production and interpretation. What (more) can VP-coordination structures tell us? *Eurosla* 13 22–46. 10.1075/eurosla.13.04pre

[B65] Rizzi L. (1986). Null objects in Italian and the theory of pro. *Linguist. Inq.* 17 501–557.

[B66] Roberts I. Holmberg A. (2010). “Parameters in minimalist theory,” in *Parametric Variation: Null Subjects in Minimalist Theory*, eds Biberauer T. Holmberg A. Roberts I. Sheehan M. (Cambridge: CUP),1–57.

[B67] Rothman J. (2009). Understanding the nature and outcomes of early bilingualism: Romance languages as heritage languages. *Int. J. Biling.* 13 145–155. 10.1177/1367006909339814

[B68] Sancier M. L. Fowler C. A. (1997). Gestural drift in a bilingual speaker of Brazilian Portuguese and English. *J. Phon.* 25 421–436. 10.1006/jpho.1997.0051

[B69] Schmid M. S. (2007). “The role of L1 use for L1 attrition,” in *Language Attrition. Theoretical perspectives*, eds Köpke B. Schmid M. S. Keijzer M. Dostert S. (Amsterdam: John Benjamins), 135–154. 10.1075/sibil.33.10sch

[B70] Schmid M. S. (2014). The debate on maturational constraints in bilingual development: a perspective from first language attrition. *Lang. Acquis.* 21 386–410. 10.1080/10489223.2014.892947

[B71] Schmid M. S. Köpke B. (2017). The relevance of first language attrition to theories of bilingual development. *Linguist. Approaches Biling.* 7 637–667. 10.1075/lab.17058.sch

[B72] Schmid M. S. Yilmaz G. (2018). Predictors of language dominance: an integrated analysis of first language attrition and second language acquisition in late bilinguals. *Front. Psychol.* 9:1306. 10.3389/fpsyg.2018.01306 30177893 PMC6110303

[B73] Seliger H. W. Vago R. M. (1991). “The study of first language attrition: an overview,” in *First Language Attrition*, eds Seliger H. W. Vago R. M. (Cambridge: Cambridge University Press), 3–16. 10.1017/CBO9780511620720.001

[B74] Sharwood Smith M. (1983). “On explaining language loss,” in *Language Development at the Crossroads*, eds Felix R. Wode H. (Tübingen: Gunter Narr Verlag), 49–59.

[B75] Silva-Corvalan C. Treffers-Daller J. (2015). *Language Dominance in Bilinguals. Issues of Measurement and Operationalization.* Cambridge, MA: Cambridge University Press.

[B76] Slavkov N. (2015). Language attrition and reactivation in the context of bilingual first language acquisition. *Int. J. Biling. Educ. Biling.* 18 715–734. 10.1080/13670050.2014.941785

[B77] Sorace A. (2005). “Selective optionality in language development,” in *Syntax and Variation*, eds Cornips L. Corrigan K. (Amsterdam: Benjamins), 55–80. 10.1075/cilt.265.04sor

[B78] Sorace A. Filiaci F. (2006). Anaphora resolution in near-native speakers of Italian. *Second Lang. Res.* 22 339–368. 10.1007/s10936-015-9372-4 25935579

[B79] Sorace A. Serratrice L. Filiaci F. Baldo M. (2009). Discourse conditions on subject pronoun realization: testing the linguistic intuitions of older bilingual children. *Lingua* 119 460–477. 10.1016/j.lingua.2008.09.008

[B80] Stolberg D. Münch A. (2010). “Die Muttersprache vergisst man nicht” – or do you? A case study in L1 attrition and its (partial) reversal. *Bilingualism* 13 19–31. 10.1017/S1366728909990332

[B81] Tsimpli I. M. (2007). “First language attrition from a minimalist perspective: interface vulnerability and processing effects,” in *Language Attrition. Theoretical Perspectives*, eds Köpke B. Schmid M. S. Keijzer M. Dostert S. (Amsterdam: John Benjamins), 83–98.

[B82] Treffers-Daller J. (2011). Operationalizing and measuring language dominance. *Int. J. Biling.* 15 147–163. 10.1177/1367006910381186

[B83] Trutkowski E. (2016). *Topic Drop and Null Subjects in German. Linguistics and Philosophy.* Berlin: De Gruyter 10.1515/9783110446173

[B84] Tsimpli I. M. (2017). Crosslinguistic influence is not necessarily attrition. *Ling. Approaches Biling.* 7 759–762. 10.1075/lab.00021.tsi

[B85] Tsimpli I. M. Sorace A. Heycock C. Filiaci F. (2004). First language attrition and syntactic subjects: a study of Greek and Italian near-native speakers of English. *Int. J. Biling.* 8 257–277. 10.1177/13670069040080030601

[B86] Unsworth S. (2015). “Amount of exposure as a proxy for dominance in bilingual language acquisition,” in *Language Dominance in Bilinguals: Issues of Measurement and Operationalization*, eds Silva-Corvalan C. Treffers-Daller J. (Cambridge: Cambridge University Press), 156–173.

[B87] Wei L. (ed.) (2007). “Dimensions of bilingualism,” in *The Bilingualism Reader.* (London: Routledge), 3–24.

[B88] White L. Genesee F. (1996). How native is near-native? The issue of ultimate attainment in adult second language acquisition. *Second Lang. Res.* 12 233–265. 10.1177/026765839601200301

[B89] Wu Y. J. Thierry G. (2010). Investigating bilingual processing: the neglected role of language processing context. *Front. Psychol.* 1:178. 10.3389/fpsyg.2010.00178 21833239 PMC3153788

[B90] Wu Y. J. Thierry G. (2013). Fast modulation of executive function by language context in bilinguals. *J. Neurosci.* 33 13533–13537. 10.1523/JNEUROSCI.4760-12.2013 23946411 PMC3742936

