# Supplementary material for: First Language Attrition and Dominance: Same Same or Different?
Source: Front Psychol. 2018 Nov 6;9:1963. doi: 10.3389/fpsyg.2018.01963 (PMC6232232; doi:10.3389/fpsyg.2018.01963)
Supplement: Supplementary file 1 [file Table_1.DOCX]

Supplementary Material

First language attrition and dominance: same same or different?

Barbara, Köpke^1^, Dobrinka, Genevska-Hanke^2^

^1^University of Toulouse, Octogone-Lordat, Toulouse, France

2 University of Oldenburg, Department of English, Oldenburg, Germany

Corresponding Author:

dobrinka.genevska.hanke@uni-oldenburg.de

# Questionnaire on Language Background for Bulgarian-German Bilinguals

(list of selected questions, content adapted for English)

1. Please list all your languages, indicating the type of each: native, second or foreign.

2. Give the age and length of acquisition for each of your second and foreign languages.

3. List all language classes/certificates you have for each of your second and foreign languages.

4. Please indicate whether you ever attended school or university abroad.

5. Indicate longer stays abroad for the countries, in which any of your second/foreign languages are spoken.

6. What language did you use most in your childhood?

7. List the languages of your mother, indicating whether these are native, second or foreign languages.

8. List the languages of your father, indicating whether these are native, second or foreign languages.

9. List the languages of your grandparents, indicating whether these are native, second or foreign languages.

10. List the languages of your siblings, indicating whether these are native, second or foreign languages.

11. List the languages of any other person you spent plenty of time with in previous times, indicating whether these are native, second or foreign languages.

12. In relation to watching TV, listening to music and phone/video calls daily, please indicate percentages for each of your languages.

12. Which languages do you speak at home?

13. Which languages do you speak at work/university?

14. Indicate how much of each language you use daily in percentages.

15. Please indicate anything else you consider important in relation to your language use.

**
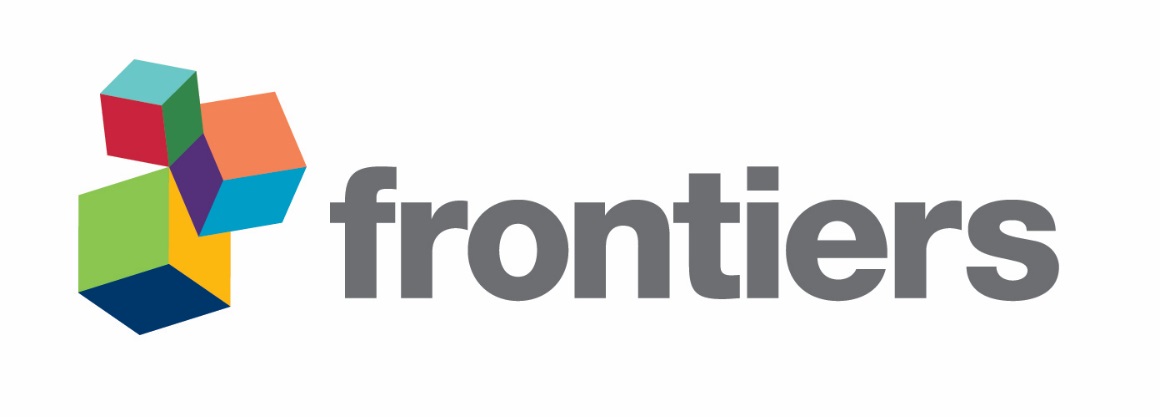
**
